# MicroRNA interactome analysis predicts post-transcriptional regulation of ADRB2 and PPP3R1 in the hypercholesterolemic myocardium

**DOI:** 10.1038/s41598-018-27740-3

**Published:** 2018-07-04

**Authors:** Bence Ágg, Tamás Baranyai, András Makkos, Borbála Vető, Nóra Faragó, Ágnes Zvara, Zoltán Giricz, Dániel V. Veres, Péter Csermely, Tamás Arányi, László G. Puskás, Zoltán V. Varga, Péter Ferdinandy

**Affiliations:** 1 Department of Pharmacology and Pharmacotherapy, Semmelweis University, 1089 Budapest, Hungary; 2 Pharmahungary Group, 6722 Szeged, Hungary; 3 Heart and Vascular Center, Semmelweis University, 1122 Budapest, Hungary; 4 Institute of Enzymology, Research Center for Natural Sciences, Hungarian Academy of Sciences, 1117 Budapest, Hungary; 5 Institute of Genetics, Biological Research Center of the Hungarian Academy of Sciences, 6726 Szeged, Hungary; 6 Department of Medical Chemistry, Semmelweis University, 1094 Budapest, Hungary; 7 Cardiovascular Research Group, Department of Biochemistry, University of Szeged, 6720 Szeged, Hungary

## Abstract

Little is known about the molecular mechanism including microRNAs (miRNA) in hypercholesterolemia-induced cardiac dysfunction. We aimed to explore novel hypercholesterolemia-induced pathway alterations in the heart by an unbiased approach based on miRNA omics, target prediction and validation. With miRNA microarray we identified forty-seven upregulated and ten downregulated miRNAs in hypercholesterolemic rat hearts compared to the normocholesterolemic group. Eleven mRNAs with at least 4 interacting upregulated miRNAs were selected by a network theoretical approach, out of which 3 mRNAs (beta-2 adrenergic receptor [*Adrb2*], calcineurin B type 1 [*Ppp3r1*] and calcium/calmodulin-dependent serine protein kinase [*Cask*]) were validated with qRT-PCR and Western blot. In hypercholesterolemic hearts, the expression of *Adrb2* mRNA was significantly decreased. ADRB2 and PPP3R1 protein were significantly downregulated in hypercholesterolemic hearts. The direct interaction of Adrb2 with upregulated miRNAs was demonstrated by luciferase reporter assay. Gene ontology analysis revealed that the majority of the predicted mRNA changes may contribute to the hypercholesterolemia-induced cardiac dysfunction. In summary, the present unbiased target prediction approach based on global cardiac miRNA expression profiling revealed for the first time in the literature that both the mRNA and protein product of Adrb2 and PPP3R1 protein are decreased in the hypercholesterolemic heart.

## Introduction

Although, many guidelines effectively support the treatment of cardiovascular disorders, cardiovascular diseases are still the leading cause of mortality and morbidity in developed countries^1^. The high morbidity and mortality of cardiovascular diseases are primarily attributed to the growing prevalence of chronic metabolic diseases, such as diabetes mellitus, hypercholesterolemia, obesity, which are well-established risk factors of numerous cardiovascular diseases (*e.g*., chronic heart failure, acute myocardial infarction, *etc*.)^1^. Hypercholesterolemia is mainly responsible for the development of atherosclerosis, therefore, hypercholesterolemic patients are exposed to more severe progression of acute and chronic ischemic heart diseases^2^. Importantly, it has been also demonstrated in preclinical and clinical studies that hypercholesterolemia directly, and independently from the development of atherosclerosis, impairs both systolic and diastolic cardiac function^3,4^, and exacerbates ischemia/reperfusion injury^5^ possibly via interfering with endogenous protective pathways^6–8^. However, the underlying mechanism of impaired myocardial function due to hypercholesterolemia is still unclear.

It is known that chronic metabolic derangements (*e.g*., type 2 diabetes mellitus) modify the microRNA (miRNA) expression pattern of fundamental metabolic and survival mechanisms of the heart^9^. MiRNAs are conserved, non-coding, about 22 nucleotide long RNA molecules, playing pivotal role in the posttranscriptional regulation of gene expression both in physiological and pathophysiological conditions^10^, including diseases of the cardiovascular system^11–13^. Generally, high-throughput miRNA analysis is applied as a screening method to identify single targets to investigate in depth. Similarly, we have previously shown that hypercholesterolemia alters miRNA expression profile significantly, and as a consequence, decreased expression of miR-25 induces oxidative/nitrative stress in the myocardium^14^. A single miRNA is able to regulate even hundreds of mRNAs, and a single mRNA might be targeted by several miRNAs, implying that a slightly changed miRNA expression profile might substantially alter multiple pathways simultaneously thereby markedly influencing the phenotype of various diseases^15,16^. Therefore, the systematic interpretation of high-throughput methods might be a powerful tool to understand the underlying complex mechanisms of cardiovascular disorders^17^.

Here we aimed to analyze the miRNA expression profile of hypercholesterolemic rat hearts with comprehensive bioinformatic methods as recommended in the Position Paper of the European Society of Cardiology Working Group on Cellular Biology of the Heart^18,19^. We developed an unbiased target prediction approach and subsequently validated changes in the expression of predicted genes, *Adrb2* and *Ppp3r1*, in case of Adrb2 direct interaction with miR-195 and miR-322 was also demonstrated.

The adrenoceptor beta 2 (*Adrb2*) gene encodes the mRNA and the protein of an adenylate cyclase-activating, G-protein coupled adrenergic receptor denoted by the symbols Adrb2 and ADRB2 respectively. While the transcribed mRNA of the protein phosphatase 3, regulatory subunit B, alpha (*Ppp3r1*) gene is denoted by the same symbol (Ppp3r1), its protein product is the calcineurin B type 1 (CNB1) which is one of the two isoforms of the regulatory subunit of the calcineurin serine/threonine phosphatase heterodimer enzyme. The mRNA (Cask) and protein product (CASK) of calcium/calmodulin dependent serine protein kinase (*Cask*) gene was also investigated in our study, which could be involved in intracellular calcium mediated signaling pathways^20^. Furthermore, gene ontology analysis revealed that an altered miRNA fingerprint is associated with impaired cardiac function and an overactivated protein kinase pattern.

## Results

### miRNA microarray analysis

**Figure 1:**
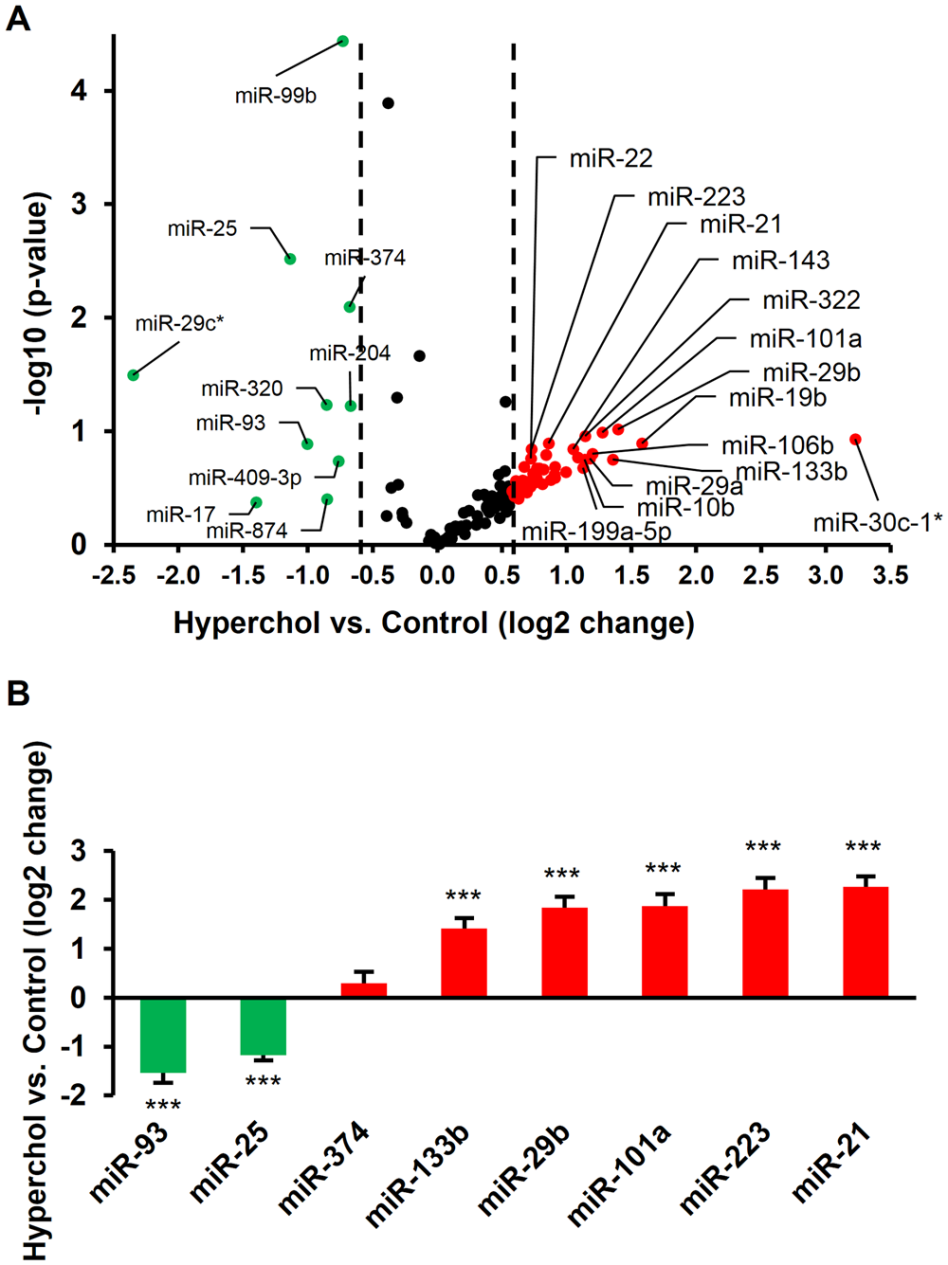
The expression difference in cardiac miRNA due to hypercholesterolemia based on miRNA microarray (**A**). The expression of selected miRNAs has been validated with qRT-PCR (**B**). ***p < 0.001 vs. Control (*ΔΔCt method*). n = 3–6/group. Downregulated and upregulated miRNAs are indicated in green and red, respectively.

Datasets of miRNA microarray analysis of normo- and hypercholesterolemic rats from our previous study^14^ were further analyzed in the present study. The miRNA microarray data were deposited in the ArrayExpress database (https://www.ebi.ac.uk/arrayexpress/) in Minimum Information About a Microarray Experiment (MIAME) compliant format with an accession number of E-MTAB-3979. Three hundred fifty miRNAs were assayed, among which 120 miRNAs were detectable. Fourty seven miRNAs were upregulated, while 10 miRNAs were downregulated in hypercholesterolemic rat hearts as compared to the normocholesterolemic control rat hearts (Fig. 1A). The expression of 8 altered miRNAs was validated with qRT-PCR (Fig. 1B).

### miRNA target prediction and validation

**Figure 2:**
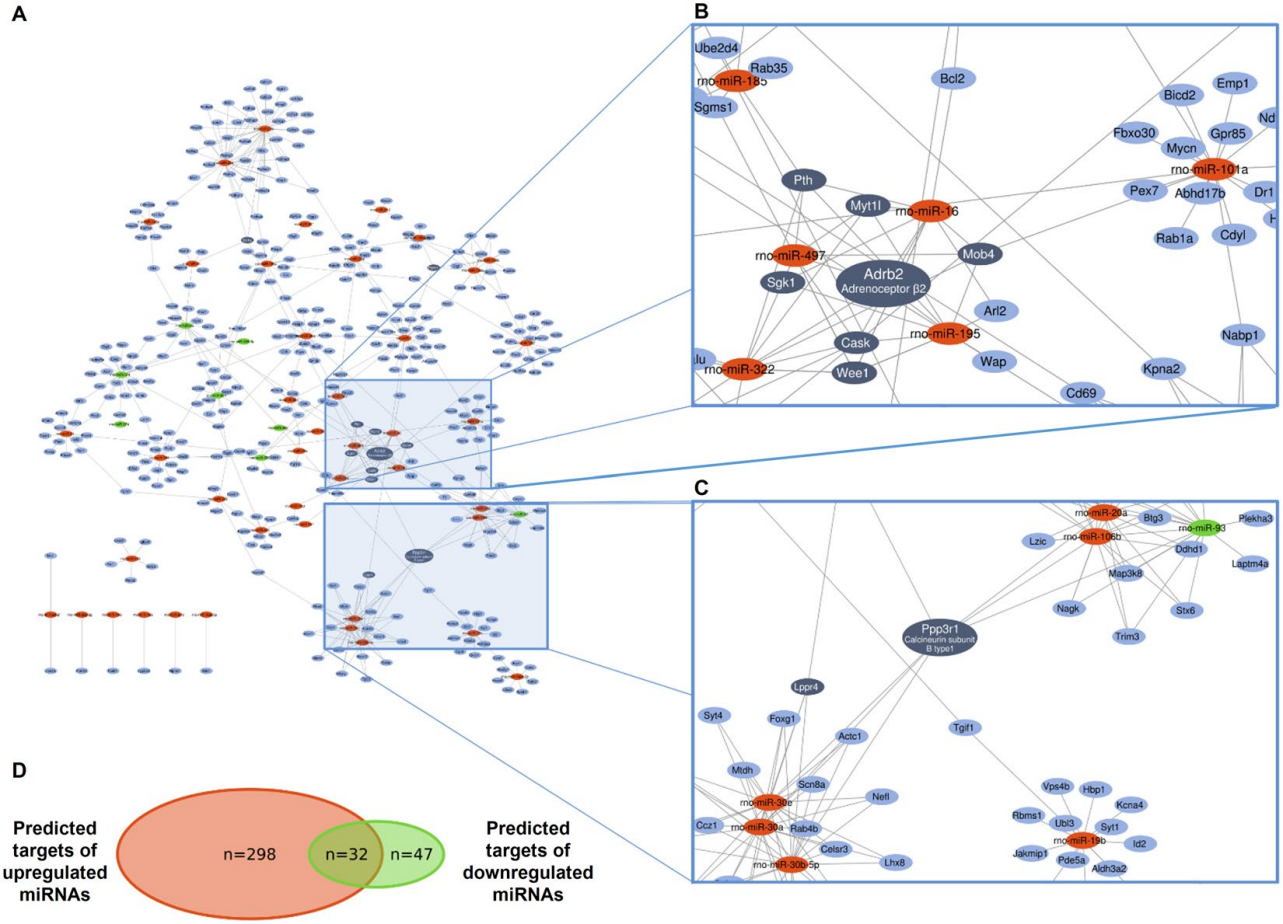
Interaction network and miRNA target prediction analysis of down- and upregulated miRNAs (**A**) showing the central role of Adrb2, Cask (**B**) and Ppp3r1 (**C**) mRNAs. High-resolution network is available as an online Supplementary material (Supplementary Fig. 1). Downregulated, upregulated miRNAs and mRNAs are indicated in green, red and blue, respectively. Dark blue represents mRNAs with at least 4 target interactions. Venn diagram presenting the number of predicted up- and downregulated miRNA targets (**D**).

Out of the differentially expressed (47 upregulated and 10 downregulated) miRNAs 43 upregulated and 8 downregulated miRNAs were further investigated. Four upregulated and two downregulated miRNAs did not meet our inclusion criteria, i.e. these miRNAs were not involved in any miRNA-target interactions presented in at least two predicted (miRDB, microRNA.org) or one experimentally validated (miRTarBase) miRNA-target interaction databases. According to previous studies, the regulation of target mRNAs by several miRNAs may be synergistic exerting a major effect on the bioavailability of the target mRNAs^21^. To find such miRNA target hubs, we constructed a classical miRNA-target network in which the nodes represent miRNAs and putative target mRNAs, while edges symbolize miRNA-target interactions (Fig. 2A; High-resolution network is available as Supplementary Fig. 1). The total number of putative mRNAs regulated by down- and upregulated miRNAs were 79 and 330, respectively, from which 32 mRNAs were theoretically modulated by both down- and upregulated miRNAs (Fig. 2D). From the indicated 409 mRNAs, 11 mRNA (Adrb2, Cask, Lppr4, Mob4, Myt1l, Ppp3r1, Pth, Ptprz1, Sgk1, Stx1a and Wee1) had at least 4 miRNA-target interactions. Out of the above 11 mRNAs four miRNA targets, i.e, Adrb2, Sgk1, Ppp3r1 and Cask mRNAs have been selected for further validation based on systematical review of the literature (Fig. 2B,C). The results of the PubMed queries are shown in Supplementary Table 1, also indicating the number of articles found to be relevant by manual curation of the search results. As parathyroid hormone (Pth) has negligible expression in the heart according to the Human Protein Atlas^22^, literature mining was not performed for Pth.

**Figure 3:**
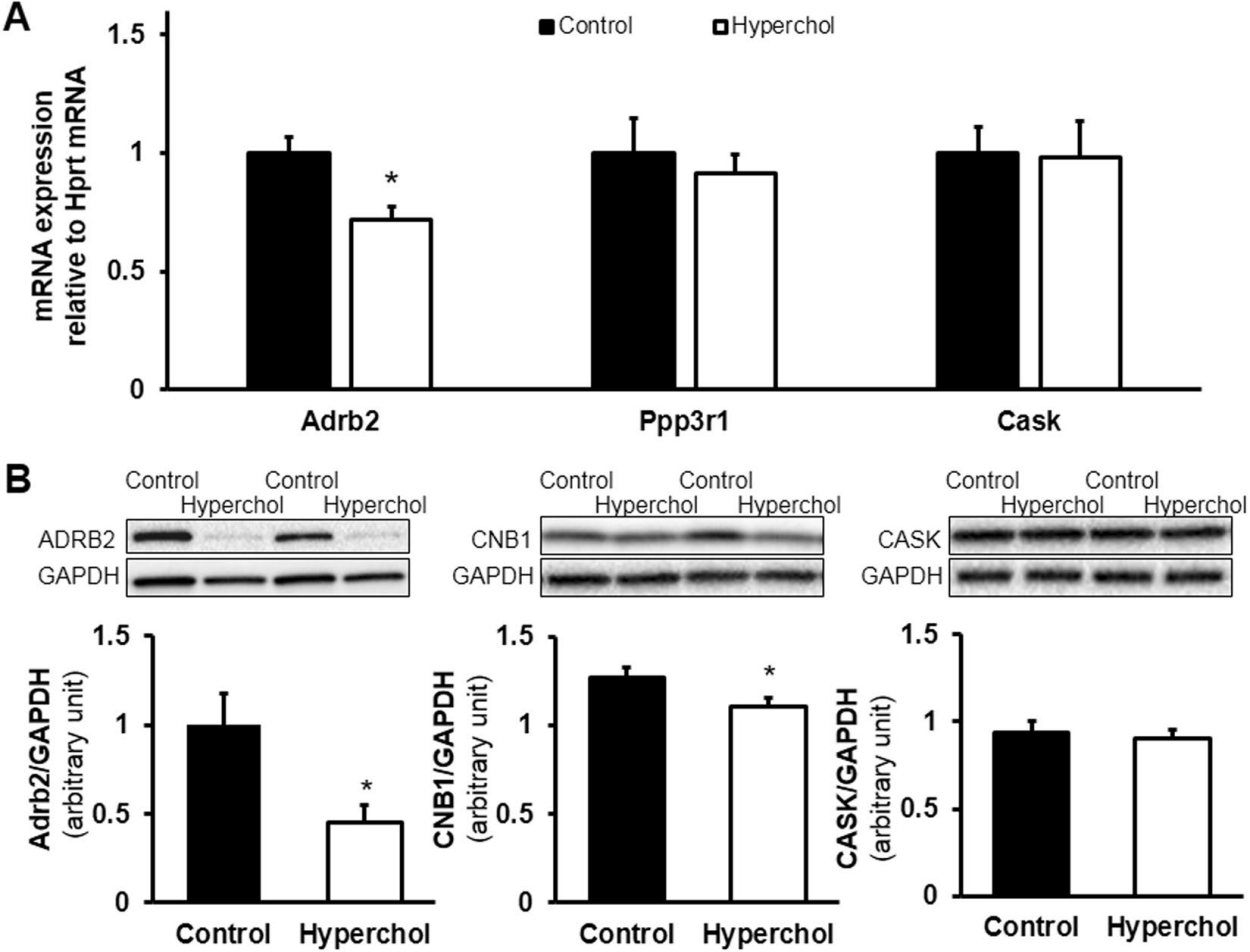
Validation of several predicted miRNA targets. Based on qRT-PCR, Adrb2 mRNA, but not Ppp3r1 and Cask mRNAs, was significantly downregulated by hypercholesterolemia in the heart compared to the control group (**A**). *p < 0.05 vs. Control (*Student’s t-test*). n = 6/group. Calcineurin B type 1 (CNB1) translated from Ppp3r1 mRNA was significantly downregulated in the hypercholesterolemic myocardium compared to the control group (**B**). *p < 0.05 vs. Control (*Student’s t-test*). n = 8–9/group. Western blot images are only representative.

The expression of Adrb2 mRNA was significantly downregulated in hypercholesterolemia compared to the normocholesterolemic group, however, mRNA expression of Ppp3r1 and Cask was not affected (Fig. 3A). Since miRNAs are not necessarily mediating the degradation of mRNAs, but also may repress their translation^23^, we assessed the expression of proteins translated from Adrb2, Ppp3r1 and Cask mRNA, i.e., adrenoceptor beta 2 (*ADRB2*), calcineurin B type 1 (CNB1) and calcium/calmodulin dependent serine protein kinase (CASK) respectively. Although cardiac *ADRB2 and* CNB1 were significantly downregulated by hypercholesterolemia, the expression of CASK was not altered by hypercholesterolemia (Fig. 3B). Full Western blot images are shown in Supplementary Fig. 2.

Out of the selected four targets we did not detect the mRNA expression of Sgk1 in our samples.

**Figure 4:**
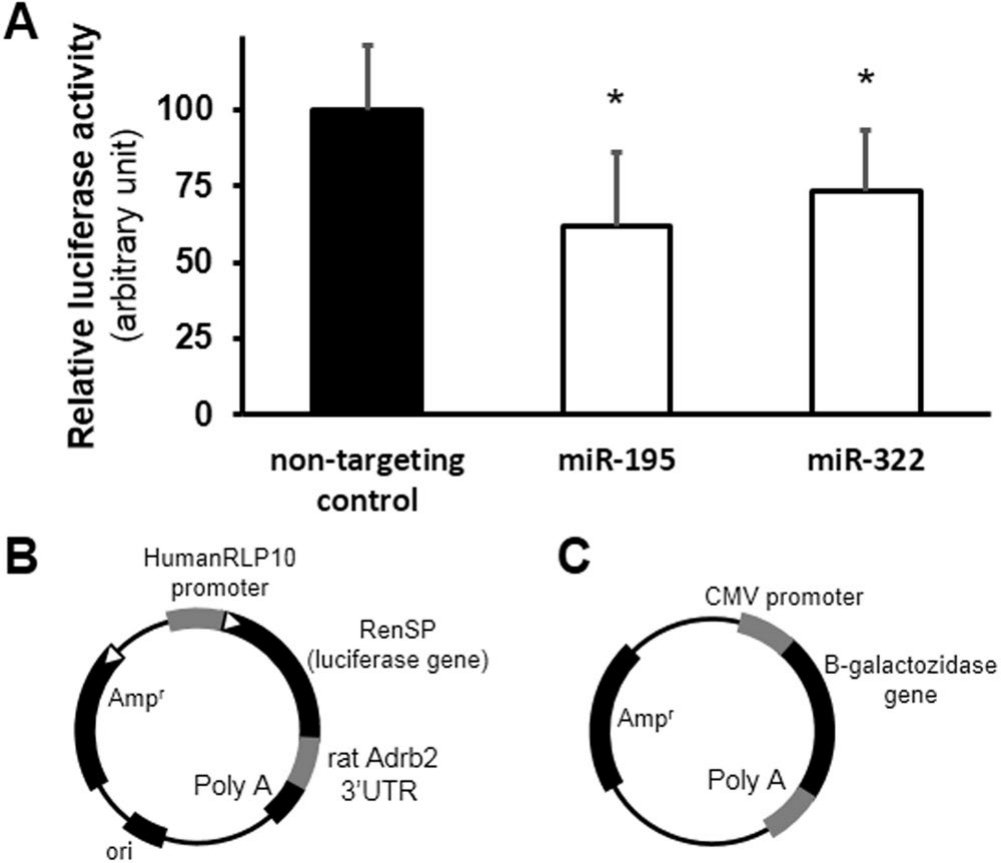
Luciferase assay validation for selected miRNAs and Adrb2 mRNA 3′UTR interaction. Luciferase signal intensity was significantly downregulated by selected targeting miRNAs, also miR-106 and miR-322, compared to non-targeting control miRNA (**A**). *<0.05 vs. non-targeting control, n = 3 (one-way ANOVA followed by Dunnett’s post hoc test) Representative maps of Lightswitch luciferase reporter vector (**B**) and beta-galactosidase reporter vector (**C**) used for miRNA luciferase assay.

The effect of chosen targeting miRNAs (miR-195, miR-322) on ADRB2 protein expression was proven in miRNA-luciferase reporter assay, constructed to show miRNA effect on predicted mRNA target. MiR-195 and miR-322 inhibited luciferase signal intensity significantly compared to signal detected after transfection with non-targeting control miRNA (Fig. 4).

### Gene ontology analysis of predicted mRNAs

**Figure 5:**
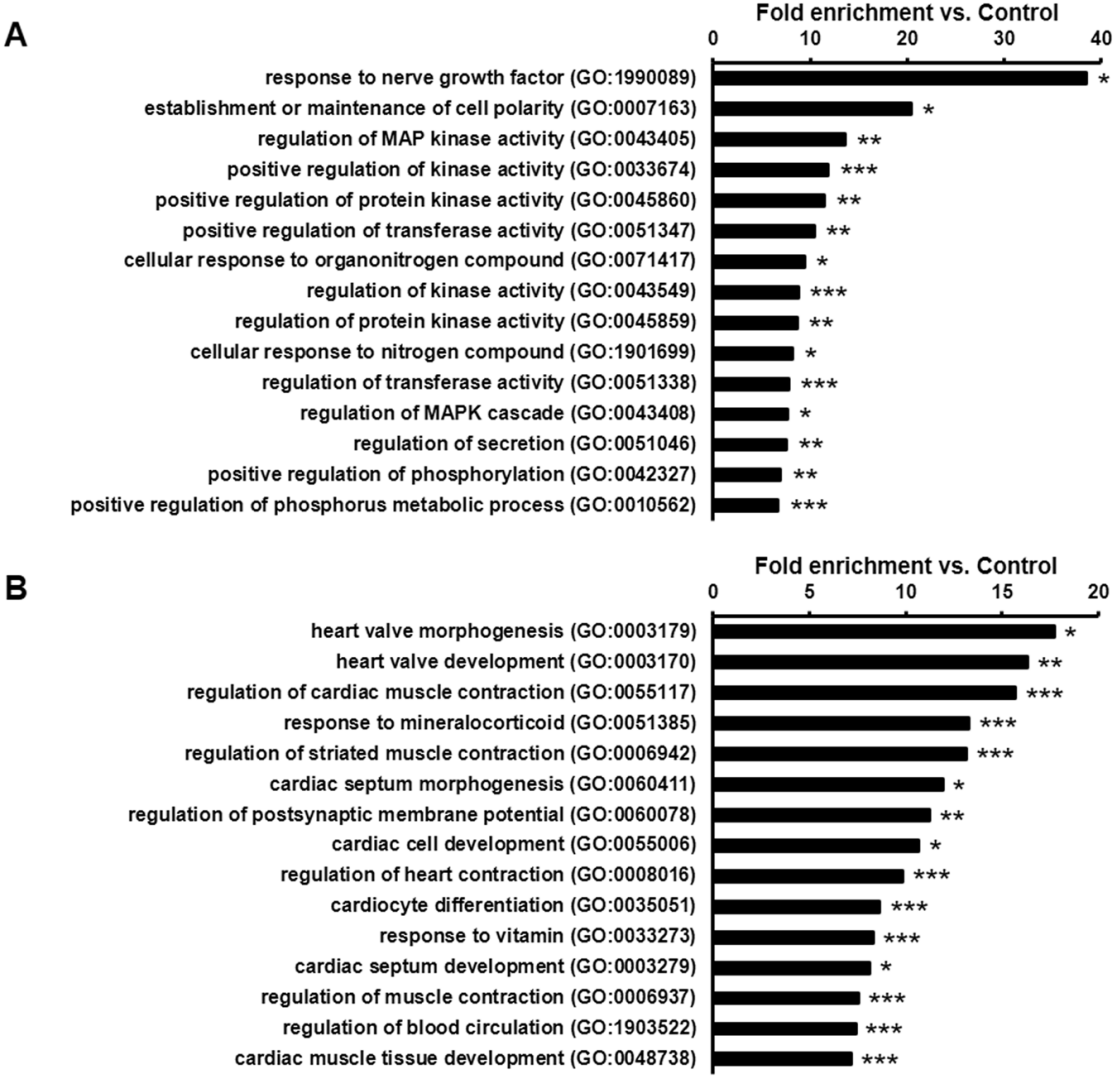
Gene ontology analysis (biological processes) of downregulated miRNA targets highlights the uncontrolled activation of mitogen-activated kinase pathways (**A**), whereas gene ontology analysis (biological processes) of upregulated miRNA targets indicates the deterioration of myocardial contractility in hypercholesterolemia (**B**). *p < 0.05, **p < 0.01, ***p < 0.001 vs. Control (Gene ontology enrichment analysis with *Bonferroni* correction).

To explore biological processes modified by hypercholesterolemia, gene ontology analysis of predicted mRNAs was performed. It clearly showed that downregulated miRNA-targeted mRNAs were significantly associated with various protein kinase activities, such as mitogen-activated protein kinases (Fig. 5A). This implies that the overall protein kinase activity may be increased in the hypercholesterolemic myocardium. Among the annotations of upregulated miRNA-targeted mRNAs, cardiac function and -development-related processes were significantly enriched and overrepresented (Fig. 5B). Similarly, developmental processes were significantly enriched among the common targets of up- and downregulated miRNAs (Supplementary Table 2). Since miRNAs hinder translation of the targeted mRNAs, gene ontology analysis indicates that cardiac function and development might be substantially impaired in the myocardium due to hypercholesterolemia.

## Discussion

In this study, we applied a bioinformatic target prediction followed by experimental target validation to identify miRNAs and related target genes differentially expressed in the heart of hypercholesterolemic rats. By comparing the miRNA expression patterns in myocardial samples from control and hypercholesterolemic rats we identified 47 and 10 candidate miRNAs to be upregulated and downregulated, respectively. The comprehensive and unbiased bioinformatic analysis of the miRNA-mRNA interactome revealed that both the mRNA and the protein product of Adrb2 gene, as well as PPP3R1 protein are decreased in hypercholesterolemia. These novel predictions were confirmed by qRT-PCR and Western blot. In case of Adrb2 gene, which showed robust expression changes on both the protein and the mRNA level, the direct interaction with miR-195 and miR-322 was verified. The present miRNA target prediction approach that contributes to the exploration of the background of hypercholesterolemia-induced cardiac dysfunction, might be a suitable tool for the global analysis and prediction of complex miRNA-mRNA interactions and may reveal relevant targets in other pathologies as well.

We have shown that hypercholesterolemia without atherosclerosis induces diastolic dysfunction^14,24,25^, which has also been verified by others^26^. Although, mechanistic details in regards of the background of hypercholesterolemia-related contractile dysfunction, such as the role of increased oxidative/nitrative stress, has been revealed^14,27^, we still do not understand the complexity of the mechanisms underlying this clinically important pathology. It has been proposed that non-coding RNAs, including miRNAs, are among the most important regulators and orchestrators of gene expression^28^. To date, most studies tended to link a particular miRNA-mediated biological effect to a single or few selected target genes. However, recent large-scale studies by using various transcriptomic and proteomic approaches, clearly demonstrated that even single individual miRNAs may alter the expression of hundreds to thousands of genes. Similarly, the mRNA transcriptome regulates the expression of miRNAs^29^, thus, organizing multiple regulatory loops. Moreover, miRNAs are also encoded in introns of mRNAs^30^ complicating the regulatory network even further. Therefore, the interactome of miRNAs and mRNAs consists of countless bidirectional connections, and change in the expression of a single RNA may profoundly influence the network dynamics. Thus, in case of significant changes in the expression of multiple miRNAs to study the overall effect on gene expression alterations, unbiased computational approaches are required. Here we hypothesized that multiple miRNAs regulating the same target would have a profound effect more likely than a single miRNA^31^. Although several computational methods have been developed and tested to date, few studies performed complex integration of target prediction of multiple miRNAs followed by experimental validation and pathway analysis. The most widely used approach is to predict targets for single miRNAs of interest and then presume biological function by performing pathway enrichment analysis on the predicted targets. Although this approach is straightforward, the predicted biological functions may not be biologically relevant if the predicted targets are not expressed by the tissues/cells under investigation.

To confirm the overall validity of our target prediction network, gene ontology analysis was performed and revealed that supposedly repressed mRNAs are significantly linked to cardiac contraction and development, which is in line with previous findings showing that hypercholesterolemia induces cardiac dysfunction^14^. Furthermore, predicted mRNA targets of downregulated miRNAs are significantly associated with the overactivation or promotion of mitogen-activated protein kinases, and it is known that various protein kinases (e.g., p38) are overactivated in hypercholesterolemia therefore inhibiting the cardiac response to various stimuli^5,32,33^.

Based on unbiased target prediction analysis, here we verified that β2-adrenoreceptor is downregulated both on the mRNA and the protein level due to hypercholesterolemia, which is associated with impaired diastolic function^14^. Previously, it has been shown that the 3′ UTR region of Adrb2 mRNA is essential to its translation^34^. Indeed, we have successfully demonstrated the direct interaction between Adrb2 mRNA and two upregulated miRNAs, namely miR-195 and miR-322, that could explain the changes in the Adrb2 expression. However, we could not rule out that other synergistic regulatory pathways (e.g., RNA-binding proteins^35^, transcription factors or epigenetic alterations^36^) are also influenced by hypercholesterolemia also contributing to the downregulation of Adrb2. Although the most abundant β-receptor in the heart is the β1-receptor, β2-receptor-related effects are also significant^37^. MiR-16 Adrb2 mRNA interaction was represented in our analysis as not validated, however this interaction was validated experimentally recently^38^. It has been shown that intravenous infusion of a selective β2 adrenergic receptor agonist, salbutamol, enhances cardiac contractility^39^, therefore, cardiac downregulation of β2-adrenoreceptor could be involved in the mechanism of myocardial dysfunction induced by hypercholesterolemia. To date, it is not known whether hypercholesterolemia represses Adrb2 mRNA expression, or if the β2 adrenoreceptor signaling abnormalities play a role in the development of hypercholesterolemia. Nevertheless, clinical studies report the β2 adrenoreceptor polymorphism as a risk factor for the development of dyslipidemia^40^. Also high LDL level is able to decrease the β2 adrenoreceptor density in porcine arteria media explants^41^, while cholesterol-depletion promotes β2-adrenerg signaling in mouse atria^42^.

Here we predicted *in silico* that Ppp3r1 mRNA is significantly suppressed by 6 upregulated miRNAs (predominantly by the miR-30 family) in hypercholesterolemia. Indeed, although the expression of Ppp3r1 mRNA was not decreased by hypercholesterolemia, the CNB1 protein (translational product of Ppp3r1 mRNA) was significantly downregulated. This is in accordance with previous findings in glomerular podocytes demonstrating that the expression of CNB1 protein is significantly decreased by miR-30 family members without affecting the mRNA level. Furthermore, the direct interaction between miR-30 family members with CNB1 mRNA was demonstrated by luciferase assays^43^. It is important to note here that although we utilized the miRNA-target interactions from miRTarBase in our software, the experimental evidence for the miRNA-target interaction between miR-30 family members and Ppp3r1 was not available in the version of miRTarBase used for the *in silico* target analysis. This literature data suggests that our predictions are in line with an independent experimental verification of the direct interaction between Ppp3r1 mRNA and the members of miR-30 family. As our microarray results indicate the upregulation of miR-30 family members in hypercholesterolemia, we consider the above experimental data from the literature sufficient to explain the observed changes in the expression of CNB1 protein. Therefore, no further luciferase reporter assays were performed in case of Ppp3r1 mRNA. It is known that the inhibition of calcineurin pathway (e.g. after renal transplantation) is associated with hypercholesterolemia^44^. However, it is not clear in our study whether the decrease in CNB1 promotes the development of hypercholesterolemia or *vice versa*. The calcineurin signaling pathway plays either an adaptive or a maladaptive role in the development of cardiac hypertrophy and heart failure^45^. It has been shown that overactivated calcineurin pathway deteriorates myocardial adaptive mechanisms, while moderately activated calcineurin, promotes cell survival, for example, following ischemia/reperfusion injury or during cardiac hypertrophy^45^. Accordingly, since calcineurin B regulates the activation of calcineurin A^46^, its downregulation due to hypercholesterolemia might contribute to cardiac diastolic dysfunction. The above literature data suggest that the expression changes of Ppp3r1 shown in our study might be involved in the development of myocardial dysfunction in hypercholesterolemia.

In case of our third predicted target, i.e. the mRNA of the *Cask* gene, we could not find significant change either in its gene or protein expression due to hypercholesterolemia. The *Cask* gene encodes a calcium/calmodulin-dependent serine protein kinase that has been described to play a role in neurons in regulating purinergic nociceptive signaling^47^ and neuronal growth^48^. Although, we found here that the rodent heart expresses Cask, Cask linked miRNAs (i.e. miR-195 and miR-322) do not seem to influence Cask expression itself. Differential expression of Cask-linked miRNAs and unaltered expression of Cask could be explained by differences in the presence of RNA binding protein sites in the predicted target RNA. RNA binding proteins, such as members of the Pumilio family, or the HuR protein family are able to bind mRNAs and affect miRNA-mediated translational repression. Pumilio members stimulate miRNA-mediated repression, while HuR proteins are capable of both supressing or facilitating (if HuR binding occurs in AU rich region) miRNA-mediated target suppression^49^. In accordance, we found consensus sequences for Pumilio binding in Ppp3r1 mRNA, explaining its downregulation. Similarly, there are AU-rich HuR binding sequences in the Adrb2 mRNA that may also explain Adrb2 downregulation^35^. However, the Cask mRNA contains HuR binding sequences that very likely inhibit miRNA-mediated silencing (by miR-195 and miR-322).

Although according to the Human Protein Atlas Sgk1 is expressed in the heart^22^, we did not detect the expression of Sgk1 on the mRNA level in our rat heart samples, suggesting species differences in the expression of this gene.

There are some limitations of our present study. Although the target prediction software we used can analyze the miRNA expression changes qualitatively (i.e. distinction between up- and downregulated miRNAs), it cannot handle those paradoxical effects that arise from the complex dynamical aspects of the miRNA mediated post-transcriptional regulation, explained in detail by the competing endogenous RNA (ceRNA) hypothesis^50^. This hypothesis can also explain our observation regarding to the internal control groups used for the fine tuning of the luciferase reporter assays (Supplementary Fig. 3). Although here we have shown experimental evidences for direct miRNA-target interactions that could explain the expression changes of Adrb2 and Ppp3r1 genes on the mRNA and protein levels, however, we can not rule out the possibility that there are miRNA-idependent regulatory pathways that could also contribute to the observed alterations in the expression of these two genes, such as changes in the activity of transcription factors, epigenetic changes and other post-transcriptional regulatory pathways^18^. If the novel targets (Adrb2 and Ppp3r1) revealed in the present study may play a role in the cardiac phenotype of this hypercholesterolemic animal model needs further investigation.

In conclusion, here we developed a comprehensive, unbiased bioinformatic method to analyze myocardial miRNA expression profile in hypercholesterolemic rats and to predict multilevel interactions of miRNAs and mRNAs and subsequently validated changes in the predicted genes, Adrb2 and Ppp3r1. We believe, that by using similar complex bioinformatic approaches, new and important molecular targets can be revealed in other clinically important pathological conditions.

## Methods

Experiments were performed according to the guidelines from Directive 2010/63/EU of the European Parliament on the protection of animals used for scientific purposes, and were approved by the local Animal Ethics Committee of the University of Szeged.

### Experimental protocol

The protocol obtaining miRNA microarray data and myocardial tissue samples of normocholesterolemic and hypercholesterolemic rats was described in detail in our previous study^14^. In the cited study, we demonstrated that hypercholesterolemia developed after feeding rats with cholesterol-enriched diet, and hypercholesterolemia leads to myocardial dysfunction. Briefly, Wistar rats were fed for 12 weeks with standard rat chow supplemented or not with 2% cholesterol and 0.25% cholate. After pentobarbital anaesthesia (60 mg/kg i.p.) and heparinization (500 U/kg i.v.), rat hearts were isolated, perfused retrogradely with Krebs-Henseleit buffer and snap frozen in liquid nitrogen. The miRNAs were isolated from ventricular myocardium (n = 6/group) according to the manufacturer’s instructions (Roche, Germany) with modifications, as described^51^. Random pairs of the RNA extracted from 6 different samples in each group were pooled, and the obtained 3 samples/group were assayed on miRNA microarrays. The expression of certain miRNAs has been validated with quantitative real-time polymerase chain reaction (qRT-PCR) as described previously, by using predesigned Taqman microRNA assays (Life Technologies, CA, US)^14^.

### Bioinformatic prediction of miRNA targets

A software (miRNAtarget.com, Pharmahungary, Szeged, Hungary) was utilized to collect those target genes that had a high probability to be regulated by differentially expressed miRNAs identified in this study. With the use of this software we queried miRNA target interactions recorded in the *Rattus norvegicus* specific subset of three publicly available databases, including two predicted (miRDB, microRNA.org) and one manually curated, experimentally validated (miRTarBase) sources^52–54^. Another highly accessed predicted database (TargetScan) was also considered as a fourth source of miRNA-target interactions, however, it did not contain data specific to rats, and thus it was excluded from the analysis^16^. Databases were downloaded as separate files between 12 and 15 January 2015 (miRDB version 5.0, released in August, 2014; microRNA.org release of August, 2010 and miRTarBase release 4.5). In the case of microRNA.org only the two bundles with good mirSVR score (a support vector regression based downregulation score ranking miRNA target sites) for both conserved and non-conserved miRNAs were used for this study. The queried miRNA-target interaction databases are not tissue specific, the predicted databases (miRDB, microRNA.org) are based on the analysis of miRNA and mRNA sequences^52–54^.

To measure the quality of predicted miRNA-target interactions, target score for miRDB and mirSVR score for microRNA.org records were checked by the software. MiRNA-target interactions with scores ≤80.0 and ≥−1.2 respectively were removed from the result set. To further reduce the ratio of false positive miRNA-target interactions, we excluded all miRNA-target interactions that were only present in one predicted database. For this purpose, we defined a compound miRNA-target interaction score giving 0.5 points for each predicted miRNA-target interaction records and 1 point for an experimentally validated record from miRTarBase. MiRNA-target interactions were kept in the resulting dataset for further analysis only if their score was greater than or equal to 1. The final output of the software was a miRNA-target network constructed from the miRNA-target interactions. The resulting network was visualized with the use of EntOptLayout plugin for Cytoscape by applying successive node position and width optimizations steps and achieving a relative entropy of 0.015^55^.

### Experimental validation of predicted miRNA targets

After target prediction, selected miRNA targets were experimentally validated by PCR, Western blotting and luciferase assay according to the recommendations of the European Society of Cardiology Working Group on Cellular Biology of the Heart^18^.

### Selection of miRNA targets for experimental validation

Predicted targets were further selected for experimental validation based on literature review of cellular pathways, possibly involved in hypercholesterolemia-induced myocardial dysfunction, i.e. alteration of calcium homeostasis, dysregulation of energy metabolism and oxidative stress. PubMed search keywords utilized for the literature mining are shown in Supplementary Table 1.

### Total RNA isolation and mRNA qRT-PCR

Total RNA was isolated from ventricular myocardium (n = 6/group) with a precipitation method. Briefly, RNAzol® RT (Sigma, MO, US) was added to each sample and homogenized with TissueLyser (Qiagen, Germany). Homogenates were centrifuged, and DNA and protein were precipitated with nuclease-free water. Furthermore, 4-bromoanisole (Sigma, MO, US), phase separation step was incorporated to maximize the DNA elimination. Total RNA was precipitated with isopropanol (vWR, PA, US), and pellets were washed twice with ethanol (vWR, PA, US). Finally, total RNA was resuspended in nuclease-free water.

Afterwards, cDNA was synthesized from total RNA (measured with NanoDrop [Thermo Fischer Scientific, MA, US]) applying Sensifast cDNA synthesis kit (Bioline, UK) according to the manufacturer’s protocol. qRT-PCR reactions were performed with a LightCycler® 480 II (Roche, Germany) in the presence of LightCycler® RNA Master SYBR Green I (Roche, Germany) and with or without dimethyl sulfoxide (DMSO; 4%; Sigma, MO, US). Primers (Integrated DNA Technologies, IA, US) were designed for surface domain of adrenoceptor beta 2 (Adrb2; forward: 5′-AACTGGTTGGGCTATGTCAA-3′; reverse: 5′-GTTAGTGTCCTGTCAGGGAG-3′), for calcineurin B type 1 (Ppp3r1; forward: 5′-CATCTCCAACCGAGACTCC-3′; reverse: 5′-GGAAAGCGAACCTCAACTTC-3′) and for calcium/calmodulin-dependent serine protein kinase (Cask; forward: 5′-TTTCAGAACCCTCCACGCT-3′; reverse: 5′-ATCTGTCTCATGTAGTGACTGG-3′). Hypoxanthine-guanine phosphoribosyltransferase (HPRT; forward: 5′-GTCCTGTTGATGTGGCCAGT-3′; reverse: 5′-TGCAAATCAAAAGGGACGCA-3′) was used as a housekeeping gene. Polymerase was activated for 15 min at 95 °C, and targets were amplified and quantified (denaturation: 30 sec at 93 °C; annealing: 30 sec at 52–53 °C; synthesis: 1 min at 72 °C).

### Western blot

Snap frozen heart samples (8 and 9 from normo- and hypercholesterolemic groups, respectively) were homogenized in radioimmunoprecipitation assay buffer (Cell Signaling Technology, MA, US) containing protease inhibitor cocktail (complete EDTA-free ULTRA Tablets, Roche, Germany; phenylmethylsulfonyl fluoride, Sigma, MO, US). Protein concentration was measured with bicinchoninic acid assay (Thermo Fischer Scientific, MA, US). Equal amounts of protein were mixed with Laemmli buffer, and were separated in 4–15% Mini-PROTEAN® TGX™ Gel (Biorad, CA, US). Proteins were transferred onto a polyvinylidene difluoride membrane (Biorad, CA, US). Membrane was blocked with Blotting-Grade Blocker (Biorad, CA, US). Membranes were incubated with primary antibodies (anti-Cask, #2878, Cell Signaling Technology, MA, US; anti-calcineurin B type 1, #AF1348, R&D Systems, MN, US; anti-beta 2 adrenergic receptor,#ab61778, Abcam, UK; anti-GAPDH, #5174, Cell Signaling Technology, MA, US), and thereafter with corresponding horseradish-peroxidase-conjugated secondary antibodies (Cell Signaling Technology, MA, US). After incubating the membranes with Clarity Western ECL Substrate (Biorad, CA, US), proteins of interest were detected with ChemiDoc XRS+ System (Biorad, CA, US). Band densities were analyzed with planimetry and compared to GAPDH.

### Luciferase assay

HeLa cells were co-transfected with luciferase vector (Lightswitch genomics, USA), beta-galactosidase vector plasmid and microRNAs (Pre-miR precursors, Thermo Fisher, FI), Lipofectamine (Thermo Fisher, FI) served as transfection agent. Luciferase vector was design to contain 3′UTR sequence of rat Adrb2 mRNA. For testing, two targeting miRNAs (miR-195 and miR-322) were chosen based of miRNA expression changes, while miR-106 served as non-targeting control. Each miRNA was used in 100 nM final concentration for transfection. After 24 hour transfection period the cells were harvested and lysed. Beta-galactosidase activity with colorimetry and luciferase activity with luminometry was measured in parallel assays. Luciferase signal intensity was normalized for beta-galactosidase signal intensity, serving as transfection control. Results are expressed in percentage of control.

### Gene ontology analysis

Gene ontology analysis was performed using all the predicted miRNA targets with the online tool of Gene Ontology Consortium (geneontology.org). Annotations of *Rattus norvegicus* species were adopted from the PANTHER Classification System (pantherdb.org) on 20 June 2016. Biological process ontology was applied to calculate enrichment.

### Statistics

qRT-PCR data were analyzed based on the *ΔΔCt method*. *Student’s t-test* was performed to analyze Western blot results. One-way ANOVA followed by Dunnett’s post hoc test was performed to analyze Luciferase results. All tests were two sided, with p < 0.05 as statistically significant level. Data were expressed as *mean* ± *standard error of mean*. Due to multiple hypotheses, *Bonferroni* correction was applied after gene ontology analysis.

### Accession codes

The miRNA microarray datasets were deposited in the ArrayExpress database (https://www.ebi.ac.uk/arrayexpress/) under the accession number of E-MTAB-3979.

## Electronic supplementary material

Supplementary Information

Supplementary Table 1

Supplementary Table 2
