# Author Correction: MicroRNA interactome analysis predicts post-transcriptional regulation of ADRB2 and PPP3R1 in the hypercholesterolemic myocardium

**DOI:** 10.1038/s41598-018-30163-9

**Published:** 2018-08-09

**Authors:** Bence Ágg, Tamás Baranyai, András Makkos, Borbála Vető, Nóra Faragó, Ágnes Zvara, Zoltán Giricz, Dániel V. Veres, Péter Csermely, Tamás Arányi, László G. Puskás, Zoltán V. Varga, Péter Ferdinandy

**Affiliations:** 1 Department of Pharmacology and Pharmacotherapy, Semmelweis University, 1089 Budapest, Hungary; 2 Pharmahungary Group, 6722 Szeged, Hungary; 3 Heart and Vascular Center, Semmelweis University, 1122 Budapest, Hungary; 4 Institute of Enzymology, Research Center for Natural Sciences, Hungarian Academy of Sciences, 1117 Budapest, Hungary; 5 Institute of Genetics, Biological Research Center of the Hungarian Academy of Sciences, 6726 Szeged, Hungary; 6 Department of Medical Chemistry, Semmelweis University, 1094 Budapest, Hungary; 7 Cardiovascular Research Group, Department of Biochemistry, University of Szeged, 6720 Szeged, Hungary

Correction to: *Scientific Reports* 10.1038/s41598-018-27740-3, published online 04 July 2018

In this Article, the ArrayExpress database accession number is incorrect. As a result, in the Results section under the ‘miRNA microarray analysis’ subheading and in the Methods section under the ‘Accession codes’ subheading,

“E-MTAB-3979”

should read:

“E-MTAB-5589”.

