# Supplementary material for: MicroRNA interactome analysis predicts post-transcriptional regulation of ADRB2 and PPP3R1 in the hypercholesterolemic myocardium
Source: Sci Rep. 2018 Jul 4;8:10134. doi: 10.1038/s41598-018-27740-3 (PMC6031673; doi:10.1038/s41598-018-27740-3)
Supplement: Supplementary file 1 — Supplementary Information [file 41598_2018_27740_MOESM1_ESM.docx]

**Supplementary Information

MicroRNA interactome analysis predicts post-transcriptional regulation of ADRB2 and PPP3R1 in the hypercholesterolemic myocardium**

Bence Ágg^a,b,c^, Tamás Baranyai^a^, András Makkos^a^, Borbála Vető^d^, Nóra Faragó^e^, Ágnes Zvara^e^, Zoltán Giricz^a^, Dániel V. Veres^f^, Péter Csermely^f^, Tamás Arányi^d^, László G. Puskás^e^, Zoltán V. Varga^a^, Péter Ferdinandy^a,b,g^

^a^ Department of Pharmacology and Pharmacotherapy, Semmelweis University, 1089, Budapest, Hungary

^d^ Pharmahungary Group, 6722, Szeged, Hungary

^c^ Heart and Vascular Center, Semmelweis University, 1122, Budapest, Hungary

^d^ Institute of Enzymology, Research Center for Natural Sciences, Hungarian Academy of Sciences, 1117, Budapest, Hungary.

^e^ Institute of Genetics, Biological Research Center of the Hungarian Academy of Sciences, 6726, Szeged, Hungary

^f^ Department of Medical Chemistry, Semmelweis University, 1094, Budapest, Hungary

^g^ Cardiovascular Research Group, Department of Biochemistry, University of Szeged, 6720, Szeged, Hungary


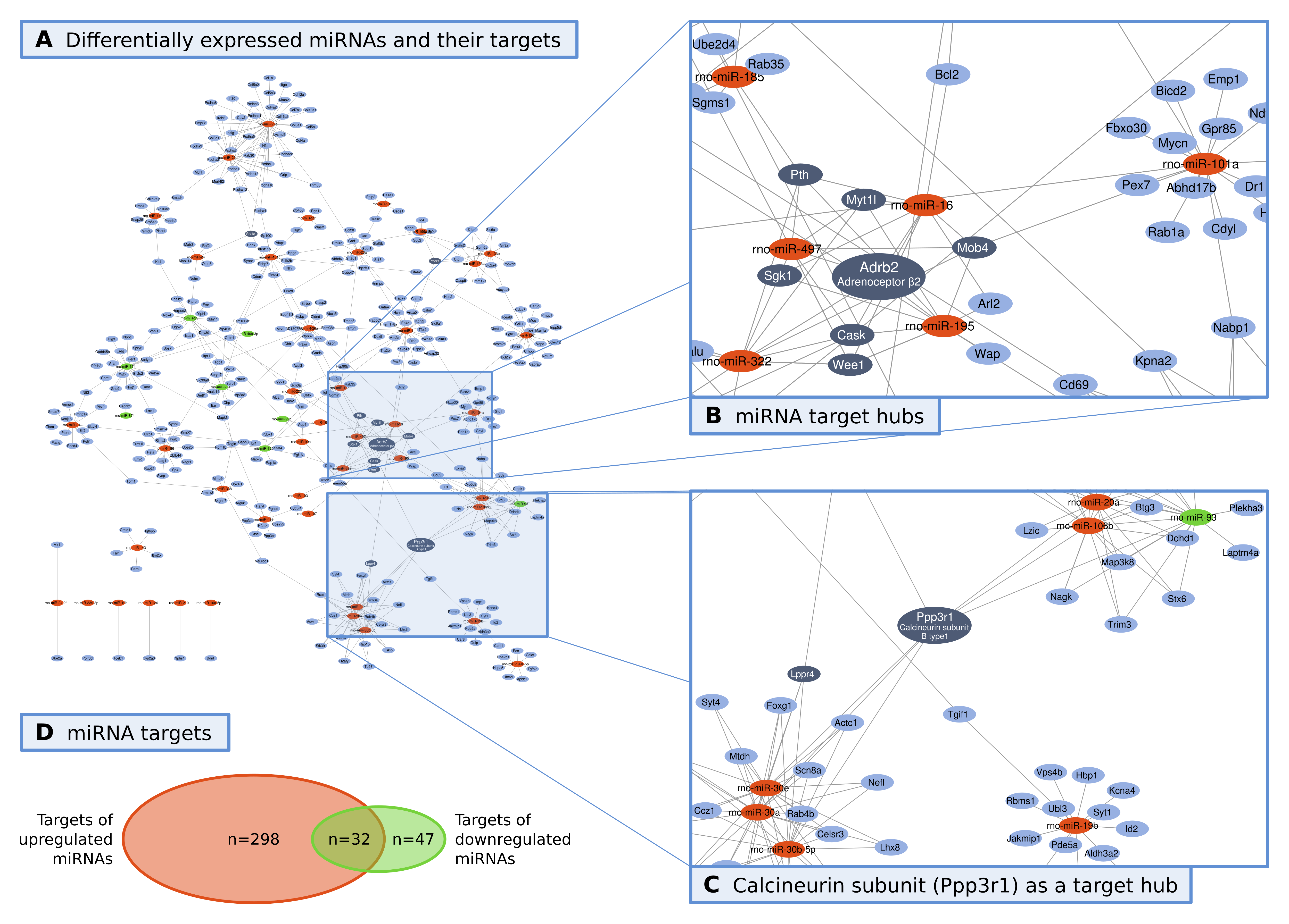


**Supplementary Figure 1.** High resolution interaction network and miRNA target prediction analysis of down- and upregulated miRNAs **(A)** showing the central role of Adrb2, Cask **(B)** and Ppp3r1 **(C)** mRNAs. Downregulated, upregulated miRNAs and mRNAs are indicated in green, red and blue, respectively. Dark blue represents mRNAs with at least 4 target interactions. Venn diagram presenting the number of predicted up- and downregulated miRNA targets **(D)**.


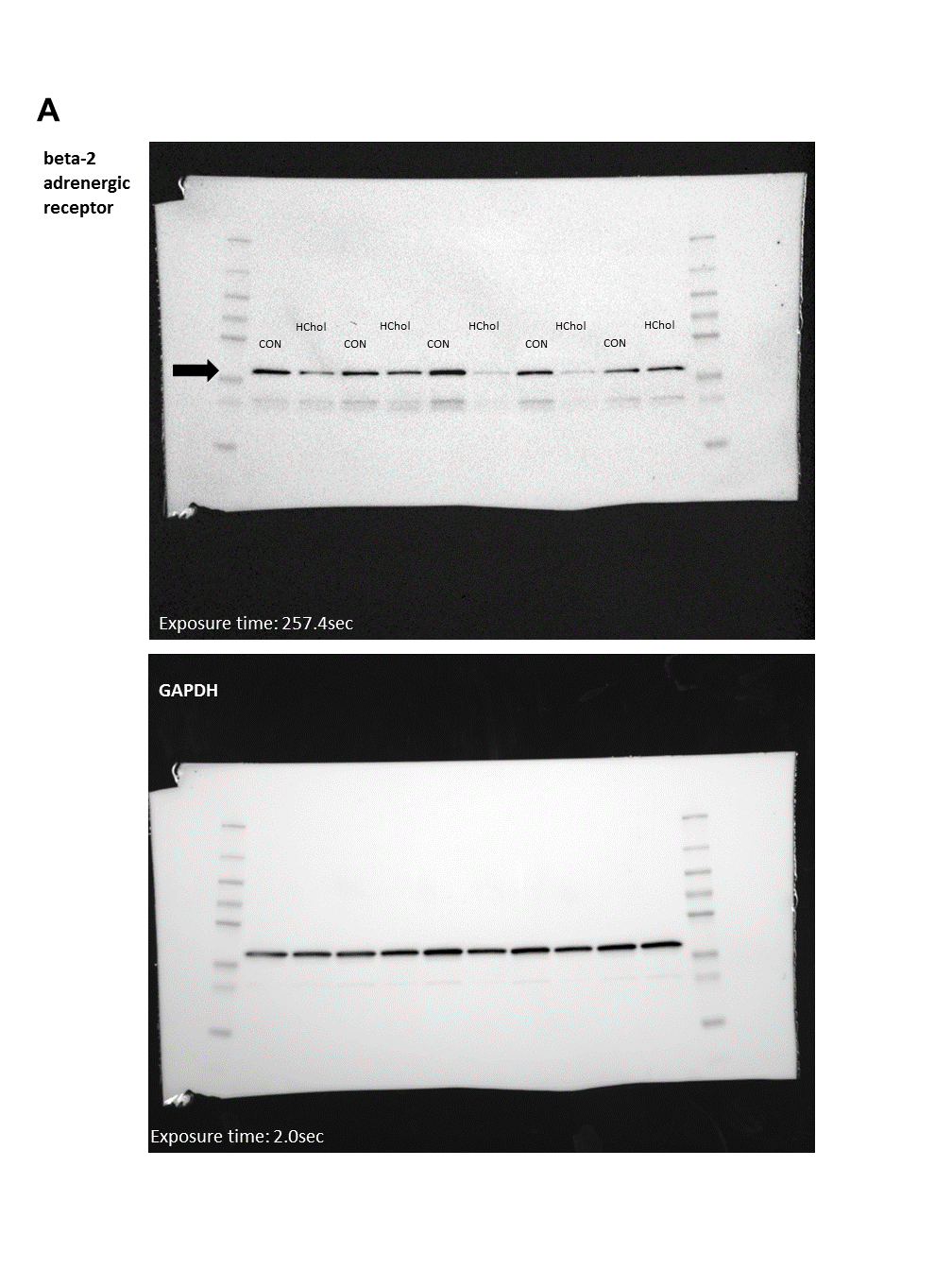


**
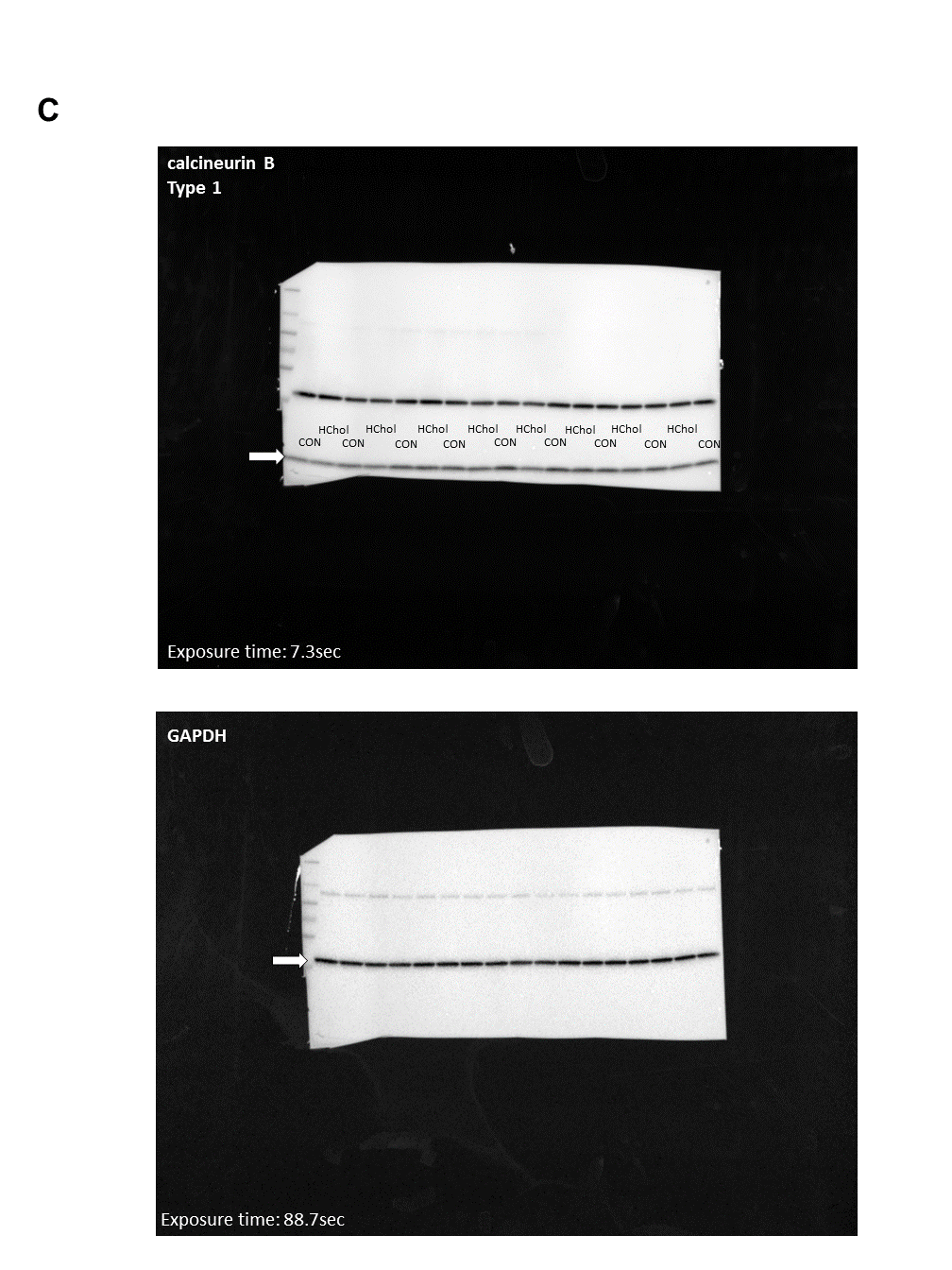
Supplementary Figure 2.** Original Western-blots of beta-2 adrenergic receptor **(A)**, calcium/calmodulin-dependent serine protein kinase **(B)** and calcineurin B **(C)** as shown in Figure 3.


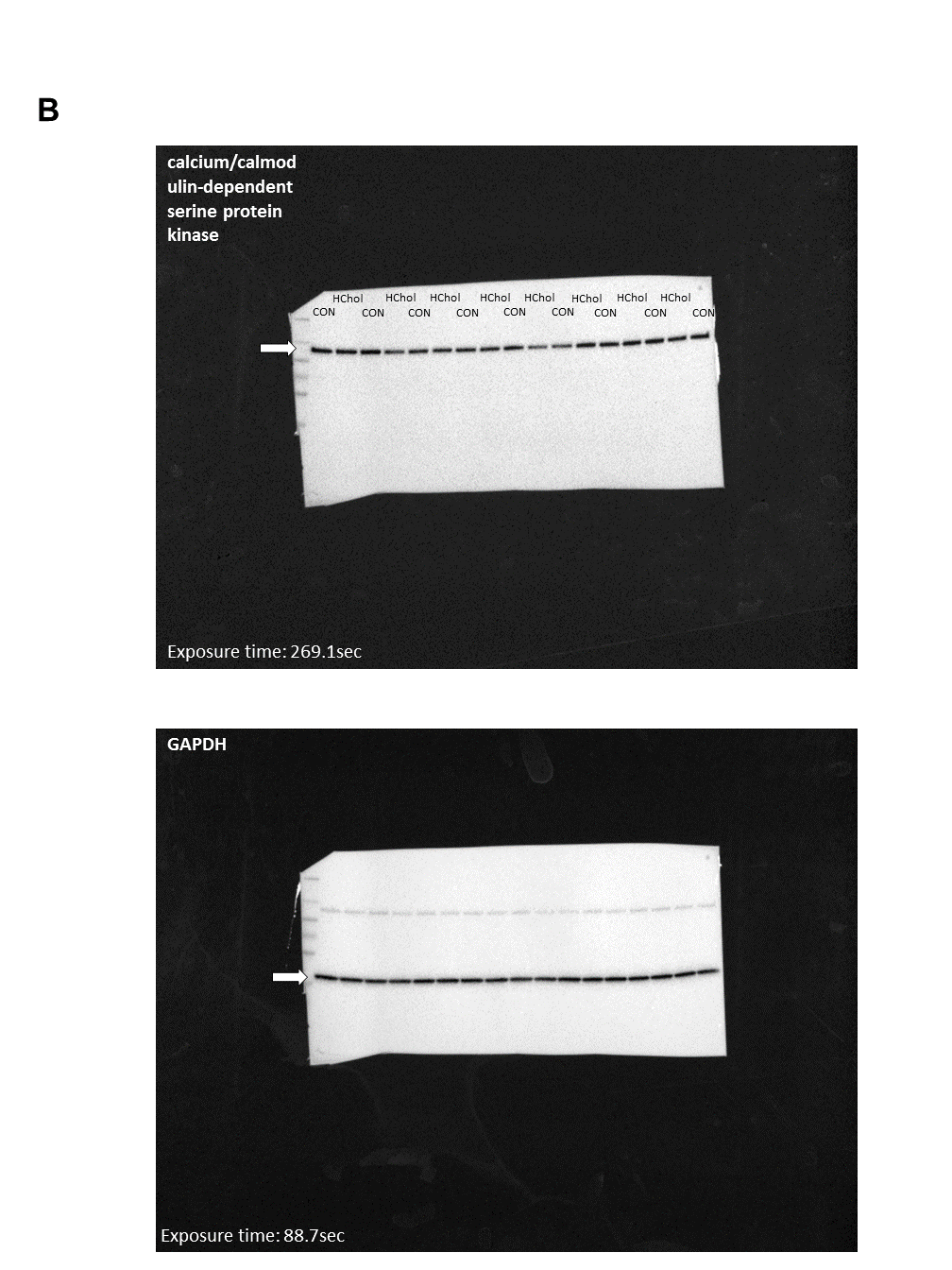


**
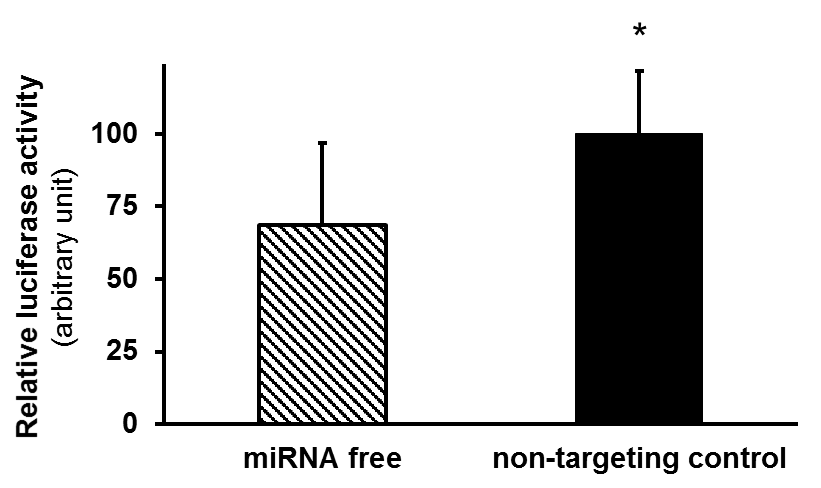
**

**Supplementary Figure 3.** In our hands luciferase assay showed higher activity when non-targeting miRNA was added compared to microRNA free internal control group.

**Supplementary Table 1.** Detailed results of literature mining performed for each predicted miRNA target. In the upper part of the table the number of items returned by PubMed and also the number of articles found to be relevant after manual curation are listed. In the lower part of the table PubMed search keywords used for the literature mining are shown. All search keyword were applied for each predicted target gene by replacing {gene} placeholder in the keyword with the symbol of the given predicted target gene.

**Supplementary Table 2.** Detailed results of gene ontology analysis performed for biological processes of targets predicted to be regulated by only downregulated, only upregulated or both down- and upregulated miRNAs in the hypercholesterolemic heart.
